# Nano-Formulating Besifloxacin and Employing Quercetin as a Synergizer to Enhance the Potency of Besifloxacin against Pathogenic Bacterial Strains: A Nano-Synergistic Approach

**DOI:** 10.3390/nano13142083

**Published:** 2023-07-16

**Authors:** Turki Al Hagbani, Syed Mohd Danish Rizvi, Shazi Shakil, Amr Selim Abu Lila

**Affiliations:** 1 Department of Pharmaceutics, College of Pharmacy, University of Ha’il, Ha’il 81442, Saudi Arabia; t.alhagbani@uoh.edu.sa (T.A.H.); a.abulila@uoh.edu.sa (A.S.A.L.); 2 King Fahd Medical Research Center, King Abdulaziz University, Jeddah 21589, Saudi Arabia; sfaruqi@kau.edu.sa; 3 Department of Medical Laboratory Technology, Faculty of Applied Medical Sciences, King Abdulaziz University, Jeddah 21589, Saudi Arabia; 4 Center of Excellence in Genomic Medicine Research, King Abdulaziz University, Jeddah 21589, Saudi Arabia; 5 Department of Pharmaceutics and Industrial Pharmacy, Faculty of Pharmacy, Zagazig University, Zagazig 44519, Egypt

**Keywords:** besifloxacin, eye infection, gold nanoparticles, quercetin, resistant pathogens, synergistic effect

## Abstract

The present study applied a nano-synergistic approach to enhance besifloxacin’s potency via nano-formulating besifloxacin on gold nanoparticles (Besi-AuNPs) and adding quercetin as a natural synergistic compound. In fact, a one-pot AuNP synthesis approach was applied for the generation of Besi-AuNPs, where besifloxacin itself acted as a reducing and capping agent. Characterization of Besi-AuNPs was performed by spectrophotometry, DLS, FTIR, and electron microscopy techniques. Moreover, antibacterial assessment of pure besifloxacin, Besi-AuNPs, and their combinations with quercetin were performed on *Staphylococcus aureus*, *Pseudomonas aeruginosa*, and *Escherichia coli*. UV-spectra showed a peak of AuNPs at 526 nm, and the electron microscopy-based size was estimated to be 15 ± 3 nm. The effective MIC_50_ concentrations of besifloxacin after loading on AuNPs were reduced by approximately 50% against the tested bacterial strains. Interestingly, adding quercetin to Besi-AuNPs further enhanced their antibacterial potency, and isobologram analysis showed synergistic potential (combination index below 1) for different quercetin and Besi-AuNP combinations. However, Besi-AuNPs and quercetin combinations were most effective against Gram-positive *S. aureus* in comparison to Gram-negative *P. aeruginosa* and *E. coli*. Their potent activity against *S. aureus* has its own clinical significance, as it is one the main causative agents of ocular infection, and besifloxacin is primarily used for treating infectious eye diseases. Thus, the outcomes of the present study could be explored further to provide better medication for eye infections caused by resistant pathogens.

## 1. Introduction

The extensive evidence of rising resistance in bacterial pathogens poses a serious clinical concern for the worldwide population. Bacterial pathogens are so sagacious that they could develop different mechanisms of resistance even toward new classes of antibiotics. To tackle these resistance issues, researchers have developed various approaches, including nano-formulations of ineffective antibiotics and adding a resistance mechanism inhibitor or synergistic molecule(s). Recently, gold nanoparticles (AuNPs) have been widely explored for the purpose of converting ineffective antibiotics into effective nano-antibiotics [1,2,3,4,5]. In fact, there are several advantages of applying AuNPs as a delivery tool, which includes the multi-valent antibacterial action of AuNPs and the ability of AuNPs to overcome most of the bacterial resistance mechanisms [6,7]. Thus, AuNPs are a delivery vehicle that could effectively deliver the antibiotic(s) or molecule(s) attached onto it, and at the same time provide an additional combat force against the bacterial pathogen. In addition, AuNPs are considered relatively less toxic than other metallic nanoparticles. However, reports suggest that AuNPs’ toxicity mainly depends on the shape, size, charge, and, most importantly, surface chemistry of AuNPs [6]. Some reports have shown that antibiotic-loaded AuNPs are non-toxic to normal human cells [4,6]. In the present study, AuNPs loaded with besifloxacin (Besi-AuNPs) were developed, characterized, and tested against different bacterial pathogens. In addition, quercetin (a natural compound) was added in combination with Besi-AuNPs and besifloxacin (alone) to observe the synergistic effect on bacterial pathogens.

Quercetin has enormous therapeutic potential against different diseases, including bacterial infections. Indeed, there are a plethora of reports that suggest the synergistic potential of quercetin when combined with antibiotics against different bacterial pathogens [8,9,10,11]. The findings suggest that quercetin in combination with antibiotics shows synergistic effects via inhibition of peptidoglycan, damaging cell membrane permeability, affecting fatty acids, deteriorating nucleic acids, decreasing the ATP level, increasing antibiotic uptake, inhibiting quorum sensing, and blocking resistance enzyme (b-lactamase) [8,9,10,12,13]. Importantly, quercetin significantly potentiates the effect of the combined antibiotic(s) against the resistant bacterial pathogens and reduces the effective dosage. Moreover, different in vivo and in vitro cytotoxicity analyses have shown that the addition of quercetin improves the safety profile of antimicrobial drugs [8,12,13,14]. Thus, the present study used quercetin as a plausible synergizer compound to evaluate its potentiating ability for besifloxacin and Besi-AuNPs against different pathogenic bacterial strains.

Besifloxacin antibiotic is a fourth-generation fluoroquinolone that is applied for ophthalmic infections. In fact, ophthalmologists usually prefer fluoroquinolones over other classes of antibiotics to treat ophthalmic infections due to their efficient ocular penetration and broad-spectrum activity. However, the rising number of resistance cases toward fluoroquinolones has raised a grave concern for the scientific community [15]. Thus, it is wiser to be prepared with an alternative option(s) rather than spending huge efforts on a newer generation of antibiotics. In the present study, besifloxacin was nano-formulated using AuNPs, and quercetin was added as a synergistic molecule to provide a better option to tackle resistance in the bacterial pathogens responsible for ophthalmic infections. It is noteworthy to mention that the applicability of both AuNPs and quercetin in ophthalmic infections is widely accepted [16,17].

The major cause of ophthalmic infections is the Gram-positive pathogen *Staphylococcus aureus*; however, some Gram-negative pathogens, i.e., *Pseudomonas aeruginosa* and *Escherichia coli*, have also been implicated in ocular infection in the past decade [18,19,20]. Hence, the prepared preparations were tested against *S. aureus*, *P. aeruginosa*, and *E. coli*. We expect to provide a formulation of besifloxacin that could be applied for ophthalmic infections with minimum concern of acquiring resistance in the near future.

## 2. Materials and Methods

### 2.1. Materials

Besifloxacin, quercetin, bacterial media, gold (III) chloride trihydrate salt, and chemicals were obtained from Sigma Aldrich (St. Louis, MO, USA).

### 2.2. Synthesis of Besi-AuNPs

A 3 mL reaction mixture was prepared by adding besifloxacin antibiotic (at 250 µg) with 1 mM gold chloride salt solution using the protocol of Alshammari et al. [4]. The reaction mixture was further kept for 48 h at 40 °C, and the change in color from light yellow to wine red visibly confirmed Besi-AuNP synthesis. The reaction mixture after the color change was centrifuged (at 30,000× *g*) for 30 min. Pellets containing synthesized Besi-AuNPs were collected and rinsed with milli-Q water followed by 50% ethanol.

### 2.3. Besi-AuNPs Characterization

Besi-AuNPs were characterized by UV-Vis spectrophotometry, dynamic light scattering (DLS), Fourier transform infrared (FTIR) spectrophotometry, and transmission electron microscopy (TEM).

#### 2.3.1. UV-Vis Spectrophotometry

A scan was performed from 250 to 700 nm using a UV-visible spectrophotometer (UV-1601, Shimadzu, Tokyo, Japan) to observe the transformation of gold (III) chloride trihydrate salt into Besi-AuNPs and the peaks of besifloxacin alone [21].

#### 2.3.2. Zeta Size and Zeta Potential Measurement by DLS

Besi-AuNPs were sonicated for 1 min and then filtered through a (0.45 µm) membrane filter prior to zeta size and potential measurement using a Malvern Nano Zetasizer (ZEN3600, Malvern Instrument Ltd., Malvern, UK). A disposable cuvette DTS0112 was used for measurement of the hydrodynamic diameter (zeta size) of Besi-AuNPs, whereas a disposable cuvette DTS1070 was used to estimate the surface zeta potential [22].

#### 2.3.3. FTIR Analysis

The binding conformation and structural changes of besifloxacin after attachment to AuNPs were observed using FTIR (Shimadzu FTIR-8201 PC, PerkinElmer Inc., Waltham, MA, USA). To attain effective signal-to-noise ratios, the diffuse (reflectance) mode was used to run the FTIR at a 4 cm^−1^ resolution. Scans of Besi-AuNPs and besifloxacin alone were recorded for a range from 400 to 4000 cm^−1^.

#### 2.3.4. TEM Analysis

Besi-AuNP samples were fixed on (carbon-coated) copper grids prior to analysis by a transmission electron microscope (Tecnai G2 Spirit) that was fitted with a BioTwin lens (Hillsboro, OR, USA). An 80 kV accelerating voltage was applied during the analysis.

### 2.4. Calculation of the Loading Efficiency of Besifloxacin on AuNPs

The besifloxacin loading amount was estimated using a UV-Vis spectrophotometer as described by Rizvi et al. [5]. The 3 mL reaction mixture after Besi-AuNP synthesis was centrifuged at 30,000× *g* for 30 min, and the supernatant was collected into a fresh tube. The concentration of unbound besifloxacin in the supernatant was estimated using a calibration curve prepared using the λmax peak [21] of pure besifloxacin at different known concentrations. Moreover, the following formula [23] was applied to calculate the percentage of loading efficiency of besifloxacin onto AuNPs:

Percentage loading efficiency of besifloxacin = [(A − B)/A] × 100(1)

where A is the initial concentration of besifloxacin added for AuNP synthesis in the reaction mixture, and B is the besifloxacin remaining or the unbound concentration in the supernatant.

### 2.5. Antibacterial Assessment

#### 2.5.1. Bacterial Strains

*Staphylococcus aureus* (ATCC 25923), *Pseudomonas aeruginosa* (ATCC 19429), and *Escherichia coli* (ATCC 25922) were procured from NCL (National Chemical Laboratory), Pune, India. A fresh culture of each bacterial strain was prepared in Luria–Bertani (LB) broth by incubating at 37 °C for 18 h. However, before experimental application, the turbidity of the bacterial culture was maintained to 0.5 McFarland standard (1.5 × 10^8^ CFU/mL).

#### 2.5.2. Qualitative Antibacterial Assessment by Well Diffusion

Qualitative assessment was performed using the agar well diffusion method [24]. The activity of Besi-AuNPs (AuNPs loaded with besifloxacin), pure besifloxacin, quercetin, a combination of quercetin with pure besifloxacin, and a combination of quercetin with Besi-AuNPs were estimated against *S. aureus*, *P. aeruginosa*, and *E. coli*. The 0.5 McFarland dilution of each strain was inoculated on Mueller–Hinton agar plates, and holes of 6 mm diameter were aseptically punched onto each plate. Then, 100 µL of Besi-AuNPs (7.2 µg/well; as determined by the loading efficiency), pure besifloxacin (7.2 µg/well), quercetin (7.2 µg/well), Besi-AuNPs (3.6 µg)/quercetin (3.6 µg), and besifloxacin (3.6 µg)/quercetin (3.6 µg) were poured into the wells. Plates after inoculation were incubated at 37 °C overnight. Triplicate experiments were performed, and an inhibition zone in millimeters was estimated as the mean ± standard deviation.

#### 2.5.3. Quantitative Antibacterial Assessment by Broth Dilution

The antibacterial effects of Besi-AuNPs, pure besifloxacin, and quercetin against *S. aureus*, *P. aeruginosa*, and *E. coli* were quantified alone and through a combinatorial approach using the broth dilution method [25]. Initially, serial dilutions of Besi-AuNPs, pure besifloxacin, and quercetin were prepared in 96-well microplates to obtain different concentrations from 2.343 to 150 µg/mL. However, combinations at a 1:1 ratio of quercetin with Besi-AuNPs and pure besifloxacin, respectively, were used to obtain a concentration range from 2.343 to 150 µg/mL. Then, 10 µL of the tested bacterial strains (1 × 10^8^ CFU/mL) was inoculated in each well and incubated for 18 h at 37 °C. The MIC_50_ was estimated for each test sample and their combination based on the minimum concentration that prevented the bacterial growth.

Furthermore, the efficiency of quercetin combined with Besi-AuNPs and quercetin combined with pure besifloxacin against the tested strains was estimated by calculating the combination index (CI) using the following formula:

CI = (D)_1_/(Dx)_1_ + (D)_2_/(Dx)_2_

where (Dx)_1_ and (Dx)_2_ are the doses for quercetin and Besi-AuNPs (or pure besifloxacin), respectively (without combination), providing 50% inhibition. Meanwhile, (D)_1_ and (D)_2_ are the doses of quercetin and Besi-AuNPs (or pure besifloxacin) in combination that showed 50% inhibition. CI > 1, CI = 1, and CI < 1 indicate antagonistic, additive, and synergistic effects, respectively [26]. Moreover, CompuSyn Version 1.0 software (Combosyn, Paramus, NJ, USA) was also applied to conduct synergy and isobologram analysis of combination [27].

#### 2.5.4. Statistical Analysis

Each experiment was run in triplicate and the outcomes are shown as the mean ± standard deviation.

## 3. Results and Discussion

**Figure 1:**
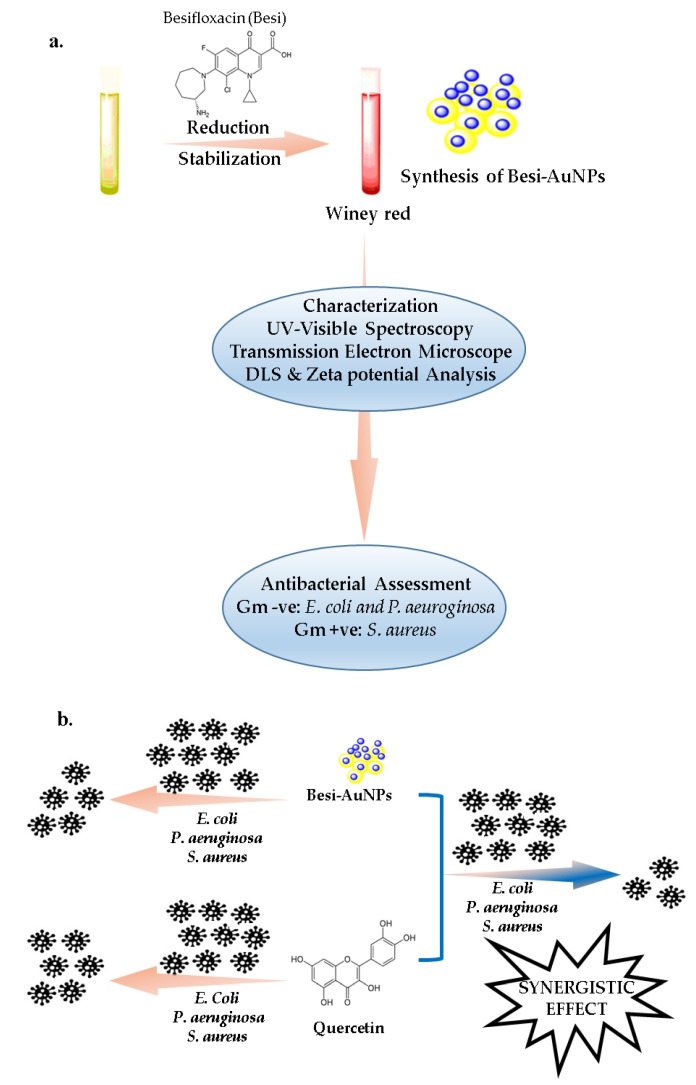
Scheme of the synthesis and characterization of Besi-AuNPs (**a**) and estimation of their antibacterial potential with and without quercetin (**b**).

The new emerging resistant strains of bacterial pathogens have raised a grave concern for the community in the past few decades. Bacterial pathogens have gained resistance toward new classes of antibiotics by different mechanisms, including efflux, uptake reduction, inactivation, and altering targets [28]. Besifloxacin is a new-generation fluoroquinolone antibiotic that shows potent activity against different pathogens responsible for ocular infections [29,30]. However, the growing ability of pathogens to acquire resistance toward fluoroquinolone antibiotics over time [15] requires researchers to be well prepared for besifloxacin resistance. Thus, in the present study, two approaches were used to increase the potency of besifloxacin: (1) Gold nano-formulations of besifloxacin were developed and (2) quercetin was added as a synergistic compound. Furthermore, these formulations were tested against the pathogens responsible for ocular infections to evaluate their potency (Figure 1).

### 3.1. Synthesis of Besi-AuNPs and Their Characterization

There are various approaches applied by researchers to synthesize AuNPs of desired sizes and to attach drugs onto their surface. However, among the different approaches, the one-pot synthesis approach, where the drug itself could synthesize and cap AuNPs, has been widely used nowadays to ease the process of drug loading and reduce the application of harmful chemicals [1,4]. Similarly, in the present study, besifloxacin was applied as a reducing and capping agent to synthesize besifloxacin-loaded AuNPs. Recently, Abu Lila et al. [2] also used a fluoroquinolone antibiotic (delafloxacin) to synthesize and cap AuNPs using the same approach. Here, piperazinyl group nitrogen of besifloxacin might have played a crucial role in the interaction with the surface of AuNPs, similar to earlier studies conducted on fluoroquinolone antibiotic-capped AuNPs [2,31,32].

#### 3.1.1. UV-Visible Spectrophotometry

**Figure 2:**
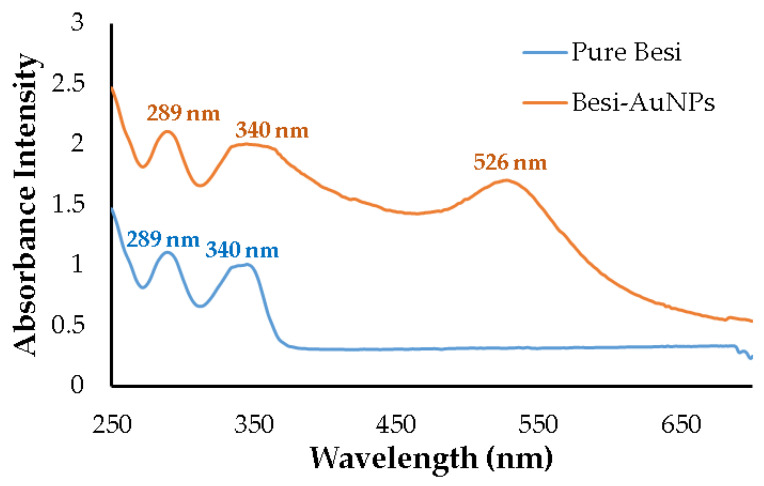
UV-visible spectrophotometry scan of Besi-AuNPs and pure besifloxacin.

AuNPs show characteristic surface plasma resonance in the visible range of 500–600 nm that can be estimated by the UV-visible spectrophotometry technique [33]. In the present study, the peak was observed at 526 nm for the synthesized Besi-AuNPs (Figure 2), which falls under the characteristic peak range, suggesting successful AuNP synthesis. Moreover, additional peaks at 289 and 340 nm were observed for besifloxacin [21], indicating the loading of besifloxacin onto AuNPs. Pure besifloxacin was also scanned to observe and confirm the peaks for the pure drug under UV-visible spectrophotometry (Figure 2). Similarly, an additional peak for delafloxacin (a fluoroquinolone antibiotic) was observed at 290 nm when loaded on AuNPs [2]. However, the color change to characteristic wine red from pale yellow also confirmed the successful synthesis of Besi-AuNPs. Thus, it could be inferred from the results that besifloxacin can effectively reduce gold salt to AuNPs and further stabilize it by capping.

#### 3.1.2. Zeta Size and Zeta Potential Measurement by DLS

**Figure 3:**
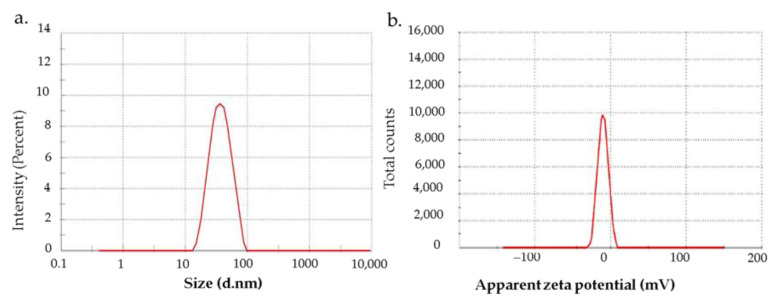
(**a**) Zeta size and (**b**) zeta potential of Besi-AuNPs.

Dynamic light scattering (DLS) is a widely applied approach to determine the size of nanoparticles and their distribution in a dispersion medium [34]. The modulation of light intensity scattered by nanoparticle dispersion was used to estimate the size of nanoparticles. Figure 3a shows the DLS results of Besi-AuNPs, depicting the hydrodynamic diameter as 62 nm. In addition, the zeta potential is another important parameter for the characterization of nanoparticles that estimates the surface charge [35]. In fact, the zeta potential represents the degree of electrostatic repulsion among the same charge particles in dispersion, indicating the stability of the colloidal preparation. Figure 3b represents the zeta potential of Besi-AuNPs, estimating it to be −12 mV, which indicates the stability of the synthesized Besi-AuNPs. The zeta potential provides information about the shield or exposure of charged surface molecules, adsorption, ionization, and distribution of nanoparticles [36]. It is suggested that particles with a higher positive/negative zeta potential repel each other and do not aggregate easily [37]. However, we cannot rely only on zeta potential data for ensuring the stability, as it is dependent mainly on repulsive electrostatic forces like Van der Waals forces. Thus, the Besi-AuNPs were visually re-examined after six months of preparation, where they were kept at room temperature. No aggregation was detected, which confirms the stable nature of the synthesized Besi-AuNPs.

#### 3.1.3. FTIR Analysis of Besi-AuNPs

**Figure 4:**
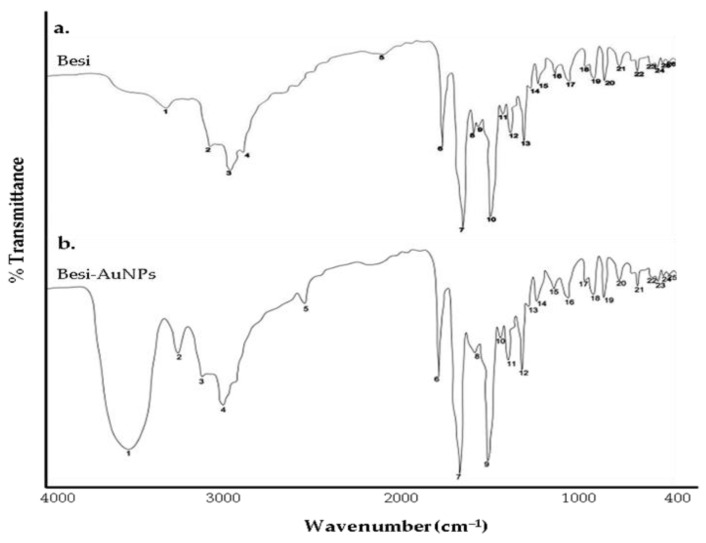
FTIR scan of (**a**) pure besifloxacin and (**b**) Besi-AuNPs.

FTIR analysis has been used in the past to identify the interaction of antibiotics/drugs with AuNPs and to confirm the efficient capping/stabilization of AuNPs [2,3,38]. In the present investigation, FTIR analysis was used to confirm besifloxacin attachment onto the surface of AuNPs. Here, pure besfloxacin and Besi-AuNPs were both subjected to scanning by FTIR to compare and confirm the attachment (Figure 4). The main peaks recorded for pure besifloxacin correspond to the functional groups –O–H stretching (3259 cm^−1^), aromatic –C–H stretching (3044 cm^−1^), aliphatic –C–H stretching (2926–2853 cm^−1^), and –C=O stretching (1609 cm^−1^), which are characteristic peaks of besifloxacin [39,40,41]. However, after Besi-AuNP synthesis, a strong –O–H stretching peak at 3449 cm^−1^ was observed, along with minor changes in the aromatic –C–H, aliphatic –C–H, and –C=O stretch peaks pertinent to besifloxacin, which suggests efficient loading of besifloxacin onto AuNPs.

#### 3.1.4. TEM Analysis

**Figure 5:**
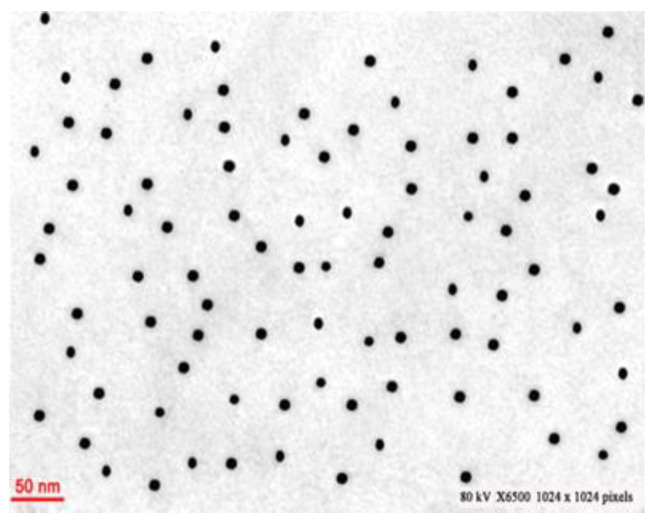
TEM micrograph image of Besi-AuNPs.

TEM analysis was used to depict the nanoparticles’ morphology, inorganic core size, and size distribution. Figure 5 shows the TEM micrograph of Besi-AuNPs, where spherically shaped monodispersed nanoparticles can be observed with an average size of 15 ± 3 nm. Interestingly, no aggregation was evident in the TEM images, indicating the effective stabilization of AuNPs by besifloxacin. However, the size by DLS was larger (62 nm) than the size suggested by TEM. These size variations were due to different principles behind the estimation of size by DLS and TEM. DLS measures the size when the sample is in a colloidal form, while TEM measures the size of the sample in a dried environment. Thus, DLS determines the hydrodynamic diameter of nanoparticles, which includes information on the dispersant solvent layer adhered to it, and TEM measures only the inorganic core without the solvent layer. A similar phenomenon of size differences of nanoparticles by TEM and DLS has been observed by several researchers in the past [3,22,38].

### 3.2. Antibacterial Assessment

**Figure 6:**
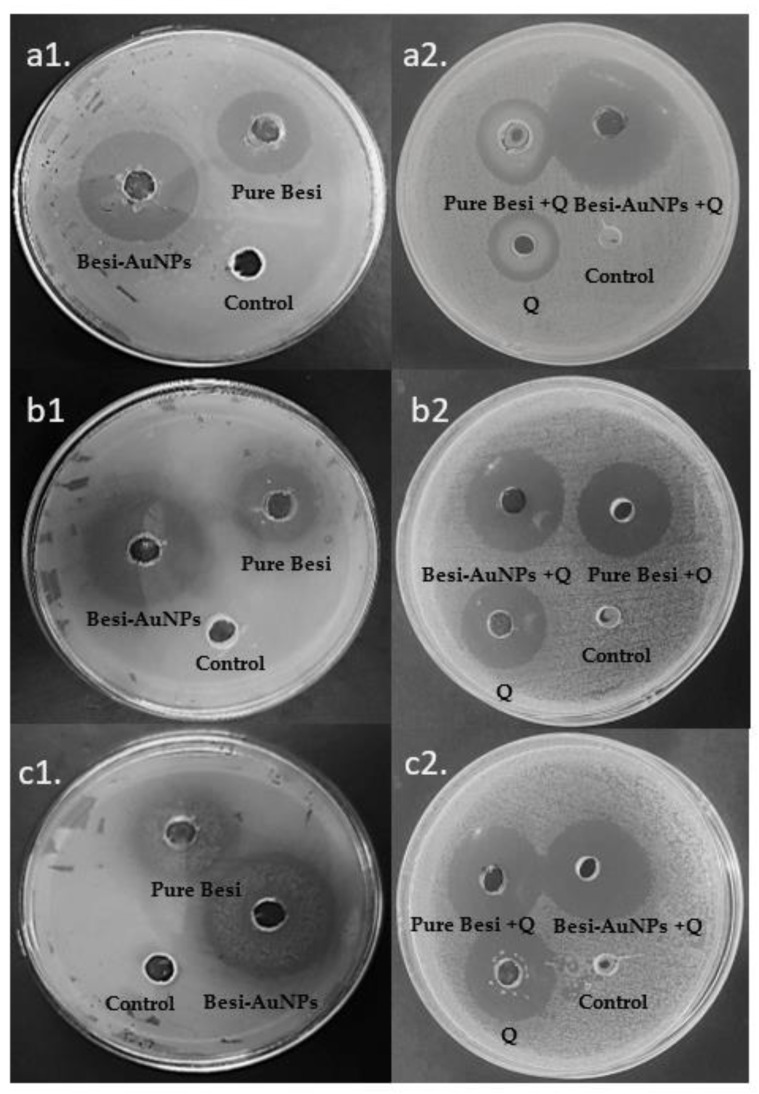
Qualitative antibacterial assessment of pure besifloxacin, Besi-AuNPs, quercetin, quercetin + pure besifloxacin, and quercetin + Besi-AuNPs against (**a1**,**a2**) *S. aureus*; (**b1**,**b2**) *P. aeruginosa*, and (**c1**,**c2**) *E. coli*.

Qualitative antibacterial assessment of Besi-AuNPs was performed on *S. aureus*, *P. aeruginosa*, and *E. coli* using the well diffusion method. The antibacterial activity of pure besifloxacin was compared with Besi-AuNPs to evaluate the potency of besifloxacin after loading to AuNPs. Moreover, quercetin was used alone and in combination (1:1 ratio) with pure besifloxacin and Besi-AuNPs, respectively, to observe the synergistic potential of quercetin. Table 1 and Figure 6 show the inhibitory potential in terms of the inhibition zone for all of the tested formulations against *S. aureus*, *P. aeruginosa*, and *E. coli*. It is evident from the results that besifloxacin after loading onto AuNPs (Besi-AuNPs) became more potent against the tested strains, and that quercetin could effectively potentiate the antibacterial effect of Besi-AuNPs and pure besifloxacin. However, the combination of Besi-AuNPs and quercetin appeared to be most potent among the tested formulations, and it showed more potency toward Gram-positive *S. aureus* (31 ± 2 mm inhibition zone) as compared to Gram-negative *P. aeruginosa* and *E. coli*. Furthermore, to confirm the qualitative findings and calculate the MIC_50_, the broth dilution approach was applied.

**Figure 7:**
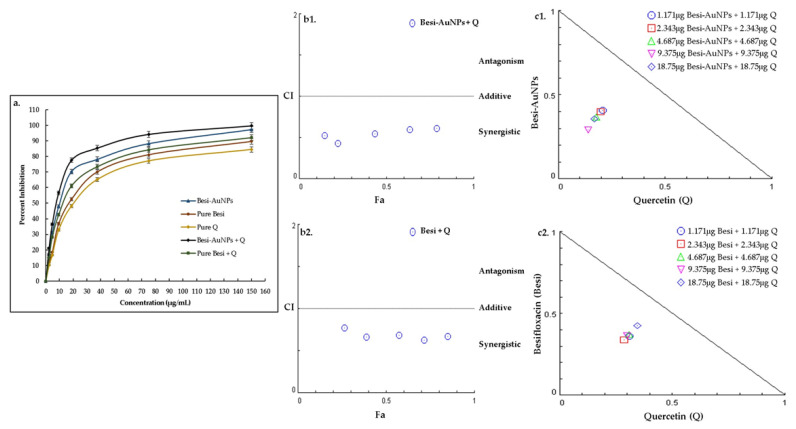
(**a**) Percentage growth inhibition of *S. aureus* after treatment with pure besifloxacin, Besi-AuNPs, quercetin, quercetin + pure besifloxacin, and quercetin + Besi-AuNPs. The experiment was repeated in triplicate, and the data shown are the mean ± standard deviation. (**b1**) Combination index plot (Fa-CI plot) of the interaction between quercetin and Besi-AuNPs against *S. aureus*. (**b2**) Combination index plot (Fa-CI plot) of the interaction between quercetin and pure besifloxacin against *S. aureus*. Here, Fa is the inhibitory effect and CI is the combination index, where, 0.5 Fa represents 50% inhibition of growth. (**c1**) CompuSyn-generated isobologram for the quercetin + Besi-AuNPs combination. (**c2**) CompuSyn-generated isobologram for the quercetin + pure besifloxacin combination. Combination data points that fall above the line are antagonistic, on the line are additive, and under the line are synergistic.

**Figure 8:**
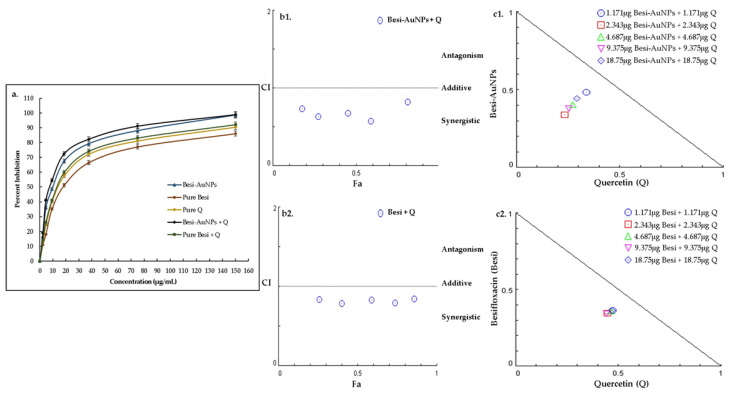
(**a**) Percentage growth inhibition of *P. aeruginosa* after treatment with pure besifloxacin, Besi-AuNPs, quercetin, quercetin + pure besifloxacin, and quercetin + Besi-AuNPs. The experiment was repeated in triplicate, and the data shown are the mean ± standard deviation. (**b1**) Combination index plot (Fa-CI plot) of the interaction between quercetin and Besi-AuNPs against *P. aeruginosa*. (**b2**) Combination index plot (Fa-CI plot) of the interaction between quercetin and pure besifloxacin against *P. aeruginosa*. Here, Fa is the inhibitory effect and CI is the combination index, where, 0.5 Fa represents 50% inhibition of growth. (**c1**) CompuSyn-generated isobologram for the quercetin + Besi-AuNPs combination. (**c2**) CompuSyn-generated isobologram for the quercetin + pure besifloxacin combination. Combination data points that fall above the line are antagonistic, on the line are additive, and under the line are synergistic.

**Figure 9:**
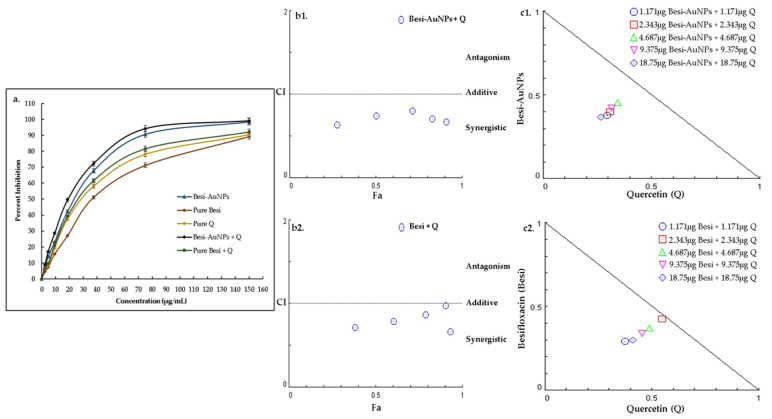
(**a**) Percentage growth inhibition of *E. coli* after treatment with pure besifloxacin, Besi-AuNPs, quercetin, quercetin + pure besifloxacin, and quercetin + Besi-AuNPs. The experiment was repeated in triplicate, and the data shown are the mean ± standard deviation. (**b1**) Combination index plot (Fa-CI plot) of the interaction between quercetin and Besi-AuNPs against *E. coli*. (**b2**) Combination index plot (Fa-CI plot) of the interaction between quercetin and pure besifloxacin against *E. coli*. Here, Fa is the inhibitory effect and CI is the combination index, where, 0.5 Fa represents 50% inhibition of growth. (**c1**) CompuSyn-generated isobologram for the quercetin + Besi-AuNPs combination. (**c2**) CompuSyn-generated isobologram for the quercetin + pure besifloxacin combination. Combination data points that fall above the line are antagonistic, on the line are additive, and under the line are synergistic.

Quantitative antibacterial assessment of besifloxacin, besifloxacin after loading on AuNPs (Besi-AuNPs), and their combination (1:1) with quercetin was carried out to calculate the MIC_50_ values against the tested strains (Table 2; Figure 7a, Figure 8a and Figure 9a). In addition, the combination index and synergistic potential of the quercetin combinations were also evaluated. Table 2 shows the MIC_50_ values of the tested formulations against *S. aureus*, *P. aeruginosa*, and *E. coli*. The results indicate that loading besifloxacin onto AuNPs reduced the effective (MIC_50_) dosage of besifloxacin by ~2 fold against the tested strains. On the contrary, quercetin further reduced the effective dosage of Besi-AuNPs and pure besifloxacin (when used in a fixed constant ratio of 1:1). Overall, Besi-AuNPs combined with quercetin showed the most potent effect compared to all other tested formulations. Moreover, *S. aureus* was found to be the most sensitive, followed by *P. aeruginosa*, and the formulations were least effective against *E. coli*.

Moreover, to confirm the synergistic effect of quercetin with Besi-AuNPs and pure besifloxacin on the tested strains, the combination index (CI) was estimated by considering the MIC_50_ data of the tested compounds alone and in combination (Table 3). For the quercetin and Besi-AuNPs combination (1:1), the CI values were estimated to be 0.573, 0.686, and 0.791 against *S. aureus*, *P. aeruginosa*, and *E. coli,* respectively. On the contrary, the quercetin and pure besifloxacin combination (1:1) showed CI values of 0.669, 0.825, and 0.793 for *S. aureus*, *P. aeruginosa*, and *E. coli,* respectively. As the value of CI for both combinations against the tested strains was below 1, the synergistic effect of the combinations is confirmed [26].

In addition, CompuSyn software was used to depict the CI plot (Fa-CI plot) of the combination against *S. aureus* (Figure 7b), *P. aeruginosa* (Figure 8b), and *E. coli* (Figure 9b). The Fa-CI plot confirmed the synergistic effect (CI < 1) of all of the tested combinations (quercetin with Besi-AuNPs and quercetin with besifloxacin) against the three tested strains. Furthermore, isobologram analysis (Figure 7c, Figure 8c and Figure 9c) also confirmed the findings, and the results depict the substantial synergistic effect of quercetin combined with Besi-AuNPs and besifloxacin against the tested strains at concentrations between 1.171 and 18.75 mg, as shown by the data points below the slope. Overall, the findings suggest that quercetin acts as a strong synergistic compound to enhance the potency of besifloxacin, either in nano-form or pure form against the tested bacterial strains.

The results of the antibacterial assessment showed that when besifloxacin is loaded on AuNPs, it could become more potent and effective, and adding quercetin could further synergistically enhance its potency. Thus, it could be inferred that the potency of besifloxacin could be enhanced by gold nano-formulating it and adding quercetin as a synergistic compound. However, a question arises about the applicability of AuNPs and quercetin in ophthalmic preparation, as besifloxacin is an antibiotic for ophthalmic infection. In fact, AuNPs have wide application in ophthalmology due to their strong stability, modulation potential, and biocompatibility [16]. They have shown promising potential as ophthalmic imaging agents because of their inertness, approachability toward the entire eye, and clearance potential from the eye [42]. An earlier investigation [43] proved that AuNPs 20 nm in size have the ability to cross the blood–retina barrier without causing any damage to the retina, and are distributed evenly in all retinal layers after intravenous injections. In a recent investigation, it was observed that surface-modulated AuNPs can easily cross the barrier of the eye and reach the retinal region, with the ability to deliver loaded drugs onto its surface [44,45]. Additionally, AuNPs appear to be a strong tool to overcome antibiotic resistance in bacterial pathogens by modulating or defying their different resistance mechanisms [6]. On the contrary, there are a plethora of reports suggesting the therapeutic potential and applicability of quercetin in ophthalmic diseases [14,46,47]. In fact, several eye drops have been formulated using quercetin as the main therapeutic molecule and tested for ocular disease treatment [46,47]. Hence, enhancing the potency of besifloxacin, either via AuNPs/quercetin or by both, has its own due clinical relevance. Interestingly, the combined formulation tested in the present study was most active against the major causative agent of eye infection, i.e., *S. aureus*. Therefore, the findings of the present study provide some hope against the increasing antibiotic resistance in the pathogens specifically responsible for eye/ocular infections. However, its toxicity features and fate in the human body are still a source of debate. It is noteworthy to mention that our research team is trying to explore the toxicity mechanism and applicable dosage via in vitro and in vivo experimental designs. The preliminary outcomes (data not shown) on some normal cell lines suggest no toxicity of Besi-AuNPs at the tested dosage concentrations. The positive outcomes prompted us to check the in vivo toxicity and toxicity parameters of the Besi-AuNPs, which is currently in progress. Thus, our team strongly hopes to develop new gold nano-formulations for ocular infection applicability in the near future.

## 4. Conclusions

In the present study, gold nano-formulations of besifloxacin (an antibiotic used for ocular infections) were developed and used in parallel with quercetin as a synergistic compound to enhance its potency against bacterial pathogens. The results showed a two-fold dose reduction after nano-formulating besifloxacin against the tested pathogens, which was further reduced when quercetin was used in combination. Notably, the besifloxacin nano-formulation and its combination with quercetin showed substantial growth inhibition of *S. aureus*, which is considered one of the major causative agents of ocular infection. Thus, the combinatorial strategy used in the present study could serve as a baseline for the preparation of a potent formulation for the treatment of eye infections caused by resistant bacterial pathogens. However, in vivo investigations are warranted before establishing it as a viable therapeutic strategy.

## Figures and Tables

**Table 1:** Zone of inhibition of besifloxacin, Besi-AuNPs, and their combination with quercetin.

Pathogen	Zone of Inhibition				
	Besi-AuNPs	Quercetin	Besi-AuNPs + Quercetin	Pure Besi	Pure Besi + Quercetin
*S. aureus*	22 ± 2 mm	17 ± 1 mm	31 ± 2 mm	16 ± 2 mm	20 ± 1 mm
*P. aeruginosa*	21 ± 2 mm	19 ± 1 mm	25 ± 2 mm	15 ± 1 mm	20 ± 2 mm
*E. coli*	20 ± 3 mm	19 ± 2 mm	23 ± 2 mm	17 ± 2 mm	21 ± 2 mm

**Table 2:** MIC_50_ values of besifloxacin, Besi-AuNPs, and their combination with quercetin.

Pathogen	MIC_50_				
	Besi-AuNPs	Quercetin	Besi-AuNPs + Quercetin	Pure Besi	Pure Besi + Quercetin
*S. aureus*	9 ± 0.9 mg/mL	19 ± 1.8 mg/mL	7 ± 0.7 mg/mL	17 ± 1.4 mg/mL	12 ± 1.3 mg/mL
*P. aeruginosa*	10 ± 1.1 mg/mL	14 ± 1.3 mg/mL	8 ± 1.1 mg/mL	18 ± 1.9 mg/mL	13 ± 1.3 mg/mL
*E. coli*	21 ± 2.1 mg/mL	28 ± 2.2 mg/mL	19 ± 1.9 mg/mL	36 ± 2.4 mg/mL	25 ± 2.2 mg/mL

**Table 3:** Combination index for quercetin combined with Besi-AuNPs and pure besifloxacin.

Pathogen	CI	
	Besi-AuNPs + Quercetin	Pure Besi + Quercetin
*S. aureus*	0.573	0.669
*P. aeruginosa*	0.686	0.825
*E. coli*	0.791	0.793

## Data Availability

Not applicable.
